# A 16-Day-Old Infant with a Clinical Diagnosis of Classical Cornelia de Lange Syndrome

**DOI:** 10.1155/2020/6482938

**Published:** 2020-04-08

**Authors:** Pamela Rodríguez, Karla Asturias

**Affiliations:** Universidad Francisco Marroquín School of Medicine, Guatemala City 01010, Guatemala

## Abstract

Cornelia de Lange syndrome (CdLS) is a rare syndromic genetic disorder characterized by multiple congenital anomalies with upper limb reduction defects, along with cardiac, gastrointestinal, and genitourinary defects. It is caused by genetic variations in the chromatin regulator genes, most commonly, the cohesin complex. Even though molecular genetic testing is highly recommended to confirm the diagnosis, high costs and unavailability in some settings are significant setbacks, and clinical criteria could be used. The typical craniofacial features include generalized hirsutism, synophrys, microbrachycephaly, highly arched eyebrows, and long eyelashes, along with height and weight below the 5^th^ percentile. In this paper, we present a case of a 16-day-old male infant in whom a clinical diagnosis of classical CdLS was made.

## 1. Introduction

Cornelia de Lange syndrome (CdLS) is a rare genetic and multisystem disorder that was initially reported by Brachmann in 1916 and described further by Cornelia de Lange in 1933, hence it is also known as Brachmann–de Lange syndrome [1, 2]. This disorder is characterized by physical, cognitive, and behavioral features that include a distinctive facial appearance, growth restriction, cognitive disabilities, behavioral problems, and limb malformations. Its prevalence is estimated to range from 1 in 10,000 to 1 in 30,000 live births [1, 3].

The syndrome has been associated with multiple abnormalities in the chromatin regulator genes, most commonly structural and regulatory genes of the cohesin complex [3, 4]. Most cases of CdLS are the result of *de novo* dominant pathogenic variants, while a minority are caused by X-linked pathogenic variants. Mutations in the *NIPBL* gene, one of the regulatory genes of cohesin, have been associated with 50–60% of the cases [1, 5].

Craniofacial features are the clinical hallmark of the syndrome. Microbrachycephaly, excess facial hair, synophrys, highly arched eyebrows, long and thick eyelashes, long philtrum, thin upper lip, depressed corners of the mouth, and low-set ears are the most notable features. Growth restriction occurs in more than 95% of patients, with height and weight usually below the 5^th^ percentile at birth and throughout life. Intellectual disability and upper limb reduction defects are also common [3, 6, 7]. Congenital heart disease, diaphragmatic hernia, and intestinal malrotation may be present, and feeding difficulties are a common management issue [2, 8].

In approximately one quarter of cases, CdLS can be suspected prenatally by ultrasonographic findings [3, 5]. Suggestive features include an increased nuchal translucency in the first trimester, low maternal serum PAPP-A level during the second trimester, intrauterine growth retardation, asymmetric upper limbs defects, congenital diaphragmatic hernia, congenital cardiac defects, and fetal abnormalities in facial profile on ultrasound scan [9]. Classical CdLS can be suspected and recognized at birth by the typical phenotype, while the milder spectrum of the disease will usually miss detection [7]. The diagnosis and the recurrence risk can be confirmed with molecular genetic testing [3]. However, these tests are not widely available in some resource-limited settings and are associated with significant economic costs. In this paper, we present a case that highlights the clinical diagnosis of a classical CdLS.

## 2. Case Presentation

A 16-day-old male presented by his mother to a primary care clinic, located in a rural area of Guatemala due to a 1-day history of umbilical redness. He was born at term via a spontaneous normal vaginal delivery to two nonconsanguineous parents, from a 32 year-old female (gravida 5, para 5), without prenatal care or ultrasounds. Birth weight was 2267 g and length of 44 cm, both values below the 3^rd^ percentile. Head circumference and APGAR score were unknown. Even though the mother recalls that the baby took several minutes to start crying, no acute complications were detected, and they were discharged from the hospital 24 hours later. Family history is remarkable for a cleft lip and cleft palate in a paternal cousin.

**Figure 1:**
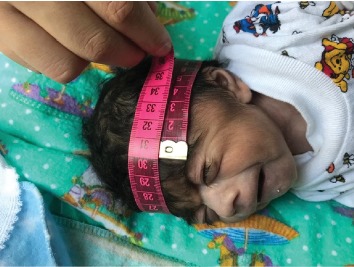
Head circumference: 30 cm (<3^rd^ percentile).

**Figure 2:**
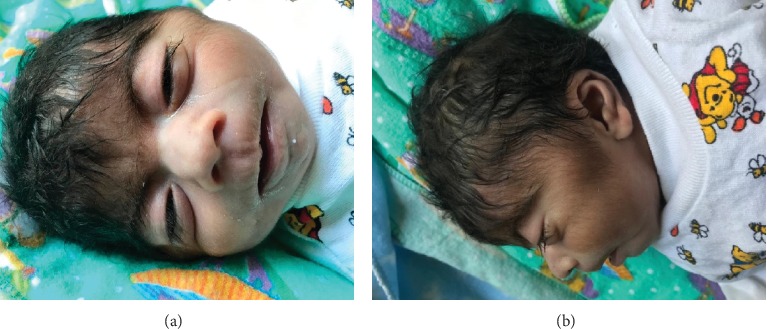
Excess facial hair, synophrys, long and arched eyebrows, long eyelashes, hypertelorism, small upturned nose, depressed and wide nasal bridge, long philtrum, thin upper lip, micrognathia, depressed corners of the mouth, and short neck.

**Figure 3:**
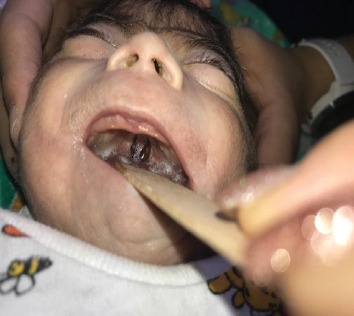
Cleft palate.

**Figure 4:**
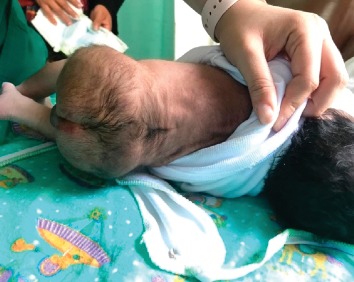
Generalized hirsutism.

**Figure 5:**
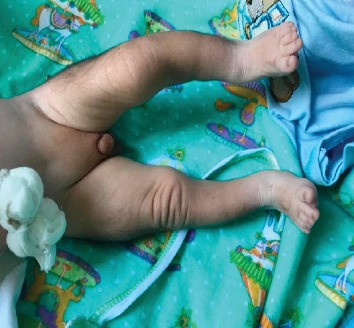
Bilateral cryptorchidism.

At the time of presentation, physical examination revealed temperature of 97.7°F (36.5°C), a heart rate of 140 bpm, respiratory rate of 38/min, pulse oximetry of 80% at room air, weight of 2170 g (<3^rd^ percentile), length of 44 cm (<3^rd^ percentile), and a head circumference of 30 cm (<3^rd^ percentile, shown in Figure 1). Craniofacial examination was notable for excess facial hair, synophrys, long and arched eyebrows, long eyelashes, hypertelorism, depressed and wide nasal bridge, a small upturned nose, low-set ears, long philtrum, thin upper lip, micrognathia, depressed corners of the mouth, and short neck (Figures 2(a) and 2(b)). Also, the patient was found to have a cleft palate (Figure 3), distal cyanosis, generalized hirsutism (Figure 4), an unhealed belly button, and bilateral cryptorchidism (Figure 5). Physical examination of the extremities was normal, with no evidence of upper limb reduction defects, congenital hand anomalies or any other major or minor limb abnormalities. Auscultation revealed a 3/6 midsystolic murmur at the upper left sternal border. An asymmetrical Moro reflex was noted, and grasp and suction reflexes were weak.

A complete blood count and a basic metabolic panel were within normal limits. A transthoracic echocardiogram revealed two atrial septal defects, consisting with both ostium primum and ostium secundum. Renal ultrasonography revealed normal findings. A karyotype was also normal for a male newborn (46, XY). The patient was clinically diagnosed with classical CdLS. Unfortunately, further genetic tests are not available in Guatemala and were not performed outside the country due to economic limitations.

## 3. Discussion

Phenotypic manifestations of CdLS can vary widely, and two main spectrums of the disease have been described: classic and mild CdLS. Classic CdLS manifests with multiple craniofacial and skeletal anomalies, growth impairment, multiorgan abnormalities, and neurocognitive delay. The milder phenotype retains many of the characteristic facial features, but with less severe cognitive and limb or structural involvement [3, 7].

Along with clinical heterogeneity, CdLS also presents with genetic heterogeneity. Multiple abnormalities in structural and regulatory genes of the cohesin complex, a ring-shaped chromatin regulator, have been described. Cohesin is an essential regulator of sister chromatid cohesion, chromosome segregation, genome integrity, telomere stability, and regulation of gene expression [3, 4]. At present, five CdLS-causative genes have been identified. Pathogenic variants in the *NIPBL* gene have been described in 50–60% of patients, with mutations that cause truncating defects (nonsense, change at splice site, and frameshift) being associated with a more severe phenotypes. Pathogenic variants in *SMC1A, SMC3, RAD21*, and *HDAC8* genes account for 5–10% of cases and are associated with mild-to-moderate phenotypes, similarly to missense variants of the *NIPBL* gene [4].

Pathogenic variants in other chromatin-associated factors have been related with disorders that overlap with CdLS, causing CdLS-like phenotypes. These genes include *BRD4, AFF4, ANKRD11, EP300, KMT2A, ARID1B, ARID1A, SMARCB1, SMARCA4, SMARCE1, ARID2, SOX11*, and *DPF2* [4].

Clinical diagnostic criteria of CdLS are based on the phenotypic appearance and includes height and weight below the 5^th^ percentile for age at birth and throughout life, limb abnormalities, mainly in the upper extremities, congenital heart defects, gastrointestinal malformations, diaphragmatic hernia, hirsutism, and synophrys. The most common limb abnormalities affect the upper extremities and include micromelia, oligodactyly, clinodactyly, and proximally placed thumbs [1, 10].

Secondary diagnostic criteria include craniofacial dysmorphisms: excess facial hair, microbrachycephaly, highly arched eyebrows, long and thick eyelashes, a wide nasal bridge, anteverted nares, a long philtrum, thin upper lip, depressed corners of the mouth, micrognathia, and low-set ears [7]. The palate is usually high, and there can be an associated cleft palate, which is often submucous. Dental anomalies have also been reported, such as small widely spaced teeth. The facial features tend to evolve as the patients grow older; the face lengthens and appears coarser and the jaw becomes more squared and bony in appearance [11].

Even though 73.1% of patients with CdLS have been described to present with limb malformations [10], our patient did not present any of them. The presence of a congenital heart defect has been described in around 25–33% of CdLS patients. The most common defects include ventriculoseptal or atrial defects, but a great variety of lesions can be present. Pulmonic stenosis, tetralogy of Fallot, and hypoplastic left heart syndrome have been described [6, 8, 11]. Cryptorchidism occurs in around 70% of males, and hypoplastic genitalia is associated in 57% of cases. Renal abnormalities, primarily vesicoureteral reflux, have been reported in 12%. Therefore, it is recommended to perform an early cardiologic and renal study, if CdLS is suspected, in order to establish an early diagnosis and treatment [3, 6, 7].

Patients are usually diagnosed after birth; however, CdLS can be suspected *in utero* by the presence of the following findings: symmetric intrauterine growth restriction, nuchal translucency in the first trimester, low maternal serum PAPP-A level during the second trimester, asymmetric upper limbs defects, congenital diaphragmatic hernia, congenital cardiac defects, and fetal abnormalities in facial profile on ultrasound scan [5, 6, 9].

At birth, the syndrome could be suspected by the presence of the typical features. Yet, due to a wide clinical phenotypic variation, milder forms of the syndrome often go unrecognized, until the first year of life [1]. Despite the fact that our patient was not recognized at birth, his early diagnosis in a primary-care setting led to the identification of associated anomalies that will lead to early interventions.

Molecular genetic testing is not always required to confirm the diagnosis; however, it can help to clarify the recurrence risk. Apparently *de novo* cases can be the result of either X-linked recessive inheritance, where confirming the mother's carrier status is important, or due to germline mosaicism in a parent, which can increase the recurrence risk for an autosomal dominant disorder. Therefore, knowing the pathogenic variant also allows prenatal diagnosis by either enhanced scanning in pregnancy or by offering invasive prenatal diagnosis by amniocentesis or chorionic villus sampling. On the downside, the cost of genetic testing can be high and is not always available, so clinical diagnostic criteria are fundamental in identifying potential individuals with the syndrome.

Medical and surgical management for CdLS is focused on the prevention of potentially treatable complications and optimization of the developmental potential [6]. Life expectancy is estimated to be 10–20 years shorter compared with the general population, depending on severity and number of complications. The most common causes of death are respiratory and gastrointestinal diseases, untreated heart defects, and seizures [11].

In conclusion, CdLS could be highly suspected with clinical findings only, and although molecular genetic testing is highly recommended, it is associated with significant costs and is not widely available in some settings, like ours. Therefore, it is important to recognize the clinical manifestations, including the craniofacial features, growth restriction, and cognitive disability, so that an early diagnosis can be made, and interventions can be performed.
